# Trabecular meshwork cells are a valuable resource for cellular therapy of glaucoma

**DOI:** 10.1111/jcmm.14158

**Published:** 2019-01-18

**Authors:** Hong Sun, Qin Zhu, Ping Guo, Yuan Zhang, Sean Tighe, Yingting Zhu

**Affiliations:** ^1^ Department of Ophthalmology the First Affiliated Hospital of Nanjing Medical University Nanjing China; ^2^ Department of Ophthalmology the Second People’s Hospital of Yunnan Province (Fourth Affiliated Hospital of Kunming Medical University) Yunnan Eye Institute, Key Laboratory of Yunnan Province for the Prevention and Treatment of Ophthalmology (2017DG008) Provincial Innovation Team for Cataract and Ocular Fundus Disease The Second People’s Hospital of Yunnan Province (2017HC010) Expert Workstation of Yao Ke (2017IC064) Kunming China; ^3^ Shenzhen University School of Medicine Shenzhen Eye Hospital Shenzhen China; ^4^ Tissue Tech, Inc. Ocular Surface Center Ocular Surface Research & Education Foundation Miami Florida

**Keywords:** glaucoma, progenitor, trabecular meshwork

## Abstract

Trabecular meshwork (TM) contains a subset of adult stem cells or progenitors that can be differentiated into corneal endothelial cells, adipocytes and chondrocytes, but not osteocytes or keratocytes. Accordingly, these progenitors can be utilized as a cell‐based therapy to prevent blindness caused by glaucoma, corneal endothelial dysfunction and other diseases in general. In this review, we review in vitro expansion techniques for TM progenitors, discuss their phenotypic properties, and highlight their potential clinical applications in various ophthalmic diseases.

## 1 GLAUCOMA

Glaucoma is a major cause of irreversible blindness worldwide and is the second leading cause of blindness in the United States.^1^ Around 80 million people in the world will suffer from this disease in 2020 and about 11 million people of those people will suffer bilateral disease.^2^, ^3^, ^4^ Glaucoma may be asymptomatic until it is too severe, thus indicating the number of glaucoma cases in the world is under‐reported.^5^ In fact, only 10%–50% people suffering from glaucoma realize they live with this condition.^6^, ^7^

Glaucoma damages the optic nerve causing progressive visual loss and blindness. Primary glaucoma is classified into two different types: open‐angle and angle‐closure. The angle refers to the angle between the iris and cornea, whereby open‐angle means the angle is wide and open. Approximately 80% cases in USA are open‐angle glaucoma and are associated with severe vision loss.^8^ Secondary glaucoma can be associated with trauma, inflammation, tumour or other medical conditions.

Elevated intraocular pressure (IOP) is one of leading risk factors of glaucoma.^9^ Experimental animal models support the notion that increased IOP may lead to optic nerve damage, similar to that of glaucoma.^10^ Current therapies to control IOP include pharmacologic agents for reduction in aqueous humour and surgical methods to increase out‐flow. These therapies are relatively effective. However, they have significant side effects, for example, toxicity, complications and other medical conditions. Despite tremendous effort by research scientists in this field, an effective cure has not been discovered as a result of the lack of understanding of the etiology of glaucoma at the molecular and cellular levels.

## 2 CHANGES OF TRABECULAR MESHWORK CELLS IN GLAUCOMA

The main out‐flow path of aqueous humour in eyes includes endothelial cell–lined channels in the anterior chamber consisting of the trabecular meshwork (TM), the collector channels, Schlemm's canal and the episcleral venous system. The TM is believed to be most resistant to aqueous out‐flow. TM cells have two major functions: assisting maintenance of aqueous out‐flow across the trabecular lamellae^12^ and secretion of extracellular matrix, specific enzymes and phagocytosis of excreted debris from the aqueous humour.^11^ Cellular dysfunction in TM is associated with aging, elevated out‐flow resistance and IOP,^13^, ^14^ suggesting that TM cells may play a critical role in maintaining normal IOP and thus preventing glaucoma. Therefore, cell‐based functional restoration of TM in eyes with glaucoma is a potential effective therapy not yet investigated because of lack of methods to sustain TM phenotype in vitro. Several studies have reported that the cells in TM retain properties of adult stem cells.^14^ However, characterization of the presumptive TM stem cells (TMSCs) is incomplete. In addition, it is unclear whether TMSCs can be expanded without loss of their phenotype, and if so, whether such expanded TMSCs can be used for regenerative therapy of glaucoma is unclear. Interestingly in glaucoma patients, the expression of TGFβ2 is abnormally higher than that from normal people, suggesting TGFβ signalling may play an important role in the progression of glaucoma.

## 3 PATHOLOGY OF TM CELLS IN GLAUCOMA

Pathological changes of TM cells in glaucoma are best described in Sihota et al.^15^ In acute primary angle‐closure glaucoma (PACG) eyes, widened spaces can be seen among the trabecular beams, with significantly increased accumulation of pigment granules under the microscopy. Phenotypically, trabecular endothelial cells are long, tapering, attenuated without regular cellular components. Elastic van Gieson staining shows a parallel distribution of the collagen and elastic components of the TM.

In chronic PACG eyes, significant alteration of the trabecular sheets with irregular trabecular spaces are observed. Endothelial cells can be observed with more immature collagen. In addition, melanin pigments are in the stroma compartment of fused trabecular beams. Under electron microscopy, the trabecular meshwork has many electron dense bodies with fibrillar‐ structured. Certain cells also contain ill‐defined vesicles without intracytoplasmic organelles, characteristic of degenerative changes.

## 4 THE PATHOGENIC ROLE OF TGF‐β IN GLAUCOMA

### 4.1 TGF‐β

The transforming growth factor‐β (TGF‐β) superfamily is a group of structurally related multifunctional regulatory proteins. The members of this superfamily include TGF‐β1‐3, BMPs and other structurally related signalling molecules.^14^ These proteins share six conserved cysteine residues required to form covalently linked dimers, which can then interact with their respective receptors.^14^ Intracellular TGF‐β signalling begins with ligand binding and subsequent activation of the TGF‐β type I receptor via phosphorylation, which leads to bridging of TGF‐β type I and II receptors on the cell membrane. This activates intracellular proteins called SMADs, which form an oligomeric complex with co‐SMAD, also an intracellular protein, to mediate the transcription of target genes and cause downstream signalling of TGF‐β.^16^, ^17^ The cellular effects of TGF‐β molecules depend on the specific TGF‐β isoform, its concentration and the target tissue.^18^ In normal physiology, cell processes influenced by TGF‐β are proliferation, recognition, differentiation and apoptosis.^17^

### 4.2 TGF‐β in aqueous humour

Numerous growth factors and cytokines are found within the aqueous humour (AH). In 1990, Granstein et al concluded that levels of TGF‐β in the AH were sufficient to serve a purpose in normal ocular physiology.^19^ Significant levels of TGF‐β in the AH of normal eyes have also been repeatedly demonstrated in animal studies.^19^, ^20^ It has been shown that TGF‐β2 is produced locally by ocular tissues and there is no correlation between AH and serum TGF‐β2 concentrations.^20^ Tripathi et al^20^ also demonstrated that porcine TM, iris and ciliary body cells express mRNA for TGF‐β2 ex vivo, and in vitro TM cells secreted the cytokine. Canine lens epithelial cells and pigmented ciliary epithelial cells in vitro also secrete TGF‐β2.^21^ Moreover, in an immune‐histochemical examination, Pasquale et al demonstrated the presence of TGF‐β in the anterior segment of normal human eyes.^22^

### 4.3 TGF‐β in different types of glaucoma

To compare the various types of glaucoma, Inatani et al^23^ showed that the level of biologically active TGF‐β2, but not total TGF‐β2, is higher in the AH of eyes with primary open angle glaucoma (POAG) than that with primary angle closure glaucoma, pseudoexfoliative glaucoma (XFG) and uveitis‐associated secondary glaucoma. Other studies have measured multiple isoforms of TGF‐β, showing that only TGF‐β2 is significantly elevated in POAG compared with non‐glaucomatous eyes, but that TGF‐β1 and TGF‐β3 are elevated in other forms of glaucoma.^24^

### 4.4 TGF‐β and IOP

Ex vivo and in vitro studies indicate that TGF‐β is involved in the pathogenesis of ocular hypertension. In an animal anterior eye segment perfusion culture, it was demonstrated that TGF‐β2 infusion for 14 days significantly increased IOP.^25^ Furthermore, TGF‐β2 antagonized the IOP‐lowering effect of interleukin (IL)‐1 in another perfusion model.^26^ Shepard et al^27^ observed an increased IOP and reduced AH out‐flow in rodents following adenoviral gene transfer of active human TGF‐β2, as well as the elevation of TM expression of TGF‐β2,^28^ which led to anatomical changes in the anterior segment that resulted in increased IOP. These studies indicate that TGF‐β is able to cause elevated IOP and is not merely an effect of elevated IOP. The TM gene expression profile also undergoes changes with IOP elevation.^29^ Vittitow and Borras^29^ revealed that in eyes with elevated IOP, genes are up‐regulated and one‐third of them are involved in signal transduction pathways. Notably, expression of matrix GLA protein (MGP) was down‐regulated by TGF‐β1. It was suggested that MGP contributes to TM tissue softening and plays a key role in maintaining pressure‐induced ocular homeostasis.^29^ This down‐regulation of MGP is another way TGF‐β may impact the extracellular cellular matrix (ECM) environment and promote conditions that elevate the IOP. Therefore, it can be assumed that patients with significantly elevated levels of TGF‐β in the AH are at an increased risk to develop elevated IOP, putting them at risk of optic nerve damage and vision loss.

### 4.5 TGF‐β, the number and phenotype of TM cells

Several growth factor receptor mRNAs, including those for TGF‐β isoforms, have been detected in cultured human TM cells and ex vivo human TM tissue from healthy and glaucomatous eyes.^30^ These receptors are densely dispersed in the TM. Cultured porcine TM cells express TGF‐β receptors with an estimated density of 4000 per cell.^31^ These studies suggest that the human eye, in particular the TM, is sensitive to TGF‐β, further implying that the elevated levels of TGF‐β seen in the AH of patients suffering from glaucoma have its biological actions in the TM. TGF‐β2 decreases the TM cell population by inhibiting cell proliferation,^30^ inducing dose‐dependent TM cell apoptosis^32^ and phagocytosis of TM cells.^33^ TGF‐β2 has also been shown to induce a secretory phenotype in cultured human TM cells by promoting collagen synthesis.^34^ This synthesis of collagen increases the ECM and may lead to TM obstruction and decreased out‐flow facility.

TGF‐β1 induces a myofibroblast‐like phenotype in human TM cells, as reflected by a dose‐dependent increase in the expression and production of α‐smooth muscle actin (αSMA) in vitro.^35^ αSMA‐positive human TM cells are spindle shaped and contain stress fibres, signifying an increase in contractility and decrease in out‐flow facility.^35^ Altered actin cytoskeletal fibres have also been shown to play a crucial role in pathogenesis of POAG and steroid‐induced glaucoma.^36^ Contrary to the previous findings, Robertson et al^28^ found that adenoviral transfer of active TGF‐β1 led to decreased αSMA. It is important to note, however, that this was TGF‐β1 isomer, and that the anatomic changes seen were more consistent with primary angle closure glaucoma as opposed to POAG.^28^ This may be one of the differences between the effects of TGF‐β1 and TGF‐β2 on the eye, and a reason that elevated TGF‐β2 is seen in POAG and TGF‐β1 seen more commonly in other forms of glaucoma.

### 4.6 TGF‐β and TM ECM turnover

TGF‐β1 and TGF‐β2 have been shown to increase ECM production and inhibit the degradation of available ECM. Gottanka et al^37^ studied the effects of TGF‐β on normal eyes in a perfused constant‐flow anterior segment organ culture model. Treatment with TGF‐β2 led to multilayered accumulation of fibrillar matrix materials under the inner wall of the canal of Schlemm and diminished the length of the canal. This resulted in diminished out‐flow facility when compared with controls, which likely correlates with increased IOPs in vivo.^37^ TGF‐β1 in vitro has been shown to increase human TM cell expression of connective tissue growth factor^38^ and elastin which could potentially contribute to out‐flow resistance.^39^

The TGF‐β2 isomer induces expression of connective tissue growth factor, thrombospondin‐1, fibronectin, collagen types IV and VI and plasminogen activator inhibitor‐1 (PAI‐1) from cultured human TM cells and bovine TM cells.^40^ TGF‐β2 also inhibits hyaluronic acid expression, most notably in the juxtacanalicular tissue.^40^ In an ex vivo model of human eye anterior segment, TGF‐β2‐induced IOP elevation was associated with increased secretion of fibronectin and PAI‐1, which was blocked by TGF‐β type I receptor inhibitors in vitro TM culture.^41^ PAI‐1 is an inhibitor of plasminogen required for the activation of matrix metalloproteinases (MMPs). Therefore, increased PAI‐1 leads to decreased activation of MMPs and may contribute to the increase in ECM of TM in glaucomatous eyes.^42^ It is also well documented that in glaucoma, MMPs are altered, as are their inhibitory enzymes, TIMPs.^43^ Therefore, the dysregulation of MMPs by increased levels of TGF‐β2 may contribute to the decreased degradation of ECM and abnormal organization commonly seen in the TM of patients with glaucoma.

### 4.7 TGF‐β and TM contraction

Contraction and relaxation of the TM cells is believed to regulate out‐flow of the AH, and thus to control IOP. It is believed that TGF‐β1 may affect the contraction of TM cells, providing another mechanism by which TGF‐β affects the IOP. In an in vitro culture of bovine TM cells in collagen gel, application of TGF‐β1 caused a dose‐dependent contraction of the collagen gel.^44^ Formation of actin stress fibres in TM cells triggered by TGF‐β1 is mediated by protein kinase C and Rho GTPase.^44^ Stress fibres are highly bundled actin filaments, which generate contractile force in the cell by connecting the cytoskeleton to the ECM. In vitro, fibronectin enhances this TM cell‐mediated collagen contraction by promoting the expression of integrin α5, thus facilitating the connection of TM cells to the surrounding matrix and the formation of stress fibres.^45^

### 4.8 Interaction of TGFβ with TM cells

TGFβ is involved in the pathogenesis of glaucoma.^41^ Higher levels of TGFβ2 in AH are associated with increased ECM content and IOP.^20^, ^23^, ^46^, ^47^ TM cells treated with TGFβ1 or TGFβ2 express ECM genes, including collagens, elastin, fibrillin, laminin and versican. Human TM cells treated with TGFβ2 also increase the level of plasminogen activator inhibitor (PAI), fibronectin, collagen and the promatrix metalloproteinase‐2.^40^, ^41^, ^48^, ^49^ In summary, TGF‐βs, especially TGF‐β2, play an important role in pathogenesis of glaucoma. It remains unclear which TGFβ signalling is activated, for example, canonical or non‐canonical TGFβ signalling in glaucoma, and if so, what is the downstream signalling in the glaucoma pathogenesis.

## 5 PROGENITOR CELLS IN TRABECULAR MESHWORK

Somatic stem cells are critical for maintenance and repair of various tissues.^50^ Adult progenitor/stem cells are present in adult bone marrow, brain, heart, skeletal muscle, limbus^52^ and trabecular meshwork.^53^ These cells are crucial for tissue renewal and can be expanded in vitro for tissue regeneration in vivo.^51^

Present evidence reveals that there is a population of progenitors in Schwalbe's Ring, the transitional area between the periphery of corneal endothelium (CE) and the anterior non‐filtering portion of the TM.^54^ This hypothesis primarily comes from the observation of an increase in TM cell division localized to the anterior non‐filtering portion of the TM after argon laser trabecuoplasty (ALT).^55^ A study has supported the notion, showing positive BrdU labelling, a marker for cell division, in the TM and posterior limbus.^56^ In order to isolate TM cell, collagenase digestion is often used, which does not disrupt cell‐cell junctions and thus retains the cell phenotype (Table 1). “Free‐floating spheres” generated from human trabecular meshwork (HTM) by collagenase digestion and cultured on a non‐adhesive substrate in Serum‐Free Expansion Medium (SFEM, a medium for in vitro culture and expansion of human haematopoietic cells) have been observed^57^ as multipotent progenitors. Stem cell markers include alkaline phosphatase, nestin and telomerase in the transition zone.^56^, ^58^

**Table 1:** The culture methods of trabecular meshwork (TM) cells

Authors and years	Tissue origin	Coat	Isolation (without T/E to single cells)	Basal medium	FBS	GFs	Phenotype	Passage and clonal growth
Du, 2012^62^	Human	None	Collagenase, clonal and conventional expansion	SCGM^a^	5%	See below	Spindle cells	FACs were used to isolate spindle cells from passage 3 TM cells and the spindle cells could be expanded up to eight passages. Only clonal expansion is effective for TM stem cell growth, which is multipotent, can be differentiated in to adipose‐like and TM cells. The TM cells can also be induced to keratocyte‐like cells only when they are cultured in keratocyte differentiation medium (advanced MEM, 10 ng/ml bFGF, 0.1 mM ascorbic acid) at the beginning, not in SCGM
Du, 2013^60^	Human	None	Collagenase, clonal culture	SCGM	5%	See below	Spindle cells	FACs were used to isolate spindle cell from passage 3 TM cells and the spindle cells could expand eight passages. Clonal expanded TM stem cells can home in
Goel, 2011^57^; 2006^64^	Human	None	Collagenase	DMEM	10%	None	N/A	N/A
Stamer, 1995^61^	Human	1% gelatin	Collagenase	Medium 199E	20%	bFGF	Broad, flat cell body with many processes	N/A
Xue, 2007^65^	Human	2% gelatin	Cut into small pieces	IMEM	P0: 20% P1‐P6: 10%	None	N/A	Pieces of TM were placed in a 2% gelatin‐coated 35‐mm dish and covered with coverslip. Cells could expand 6 passages
Yu, 2008^85^	Human	None	Cut into small pieces	DMEM	10%	None	N/A	Pieces of TM were placed in sterile 35 mm petri dishes and kept in with a coverslipPassages not available

a Stem cell growth medium (SCGM): modified from a corneal endothelial cell culture medium that contained reduced serum medium (OptiMEM‐1) supplemented with 5% foetal bovine serum; 10 ng/mL EGF; 100 μg/mL bovine pituitary extract; 20 μg/mL ascorbic acid; 200 μg/mL calcium chloride; 0.08% chondroitin sulfate; 100 IU/mL penicillin; 100 μg/mL streptomycin and 50 μg/mL gentamicin.

A report suggested that progenitors from human TM expanded in vitro showed their ability to differentiate into TM cells in vivo in mice. In this study, DiO‐labelled TMSCs injected into the anterior chamber of normal mice were localized primarily in TM, remaining in the tissue at least 4 months. In 1 week, TMSCs expressed TM marker CHI3L1. Almost no apoptosis could be seen in injected TM tissue and IOP was not increased in the experiment.^60^ In another study, Nadri et al have isolated a group of mesenchymal stem cells from human TM, which can be differentiated into photoreceptor‐like cells on amniotic membrane.^59^

However, it is unclear how the trabecular meshwork progenitors can be maintained and expanded in vitro without change in their phenotype. In addition, it is also unclear whether TM progenitors can differentiate into corneal endothelium (CE), fat and cartilage, as we know they may have or may not have a similar origin and a close anatomical position. Thus, the potential to repair or replace the human corneal TM cells, to generate endothelial or other tissues by TM progenitors is also an important area that needs to be explored.

## 6 2‐D matrigel as the culture substrate for TM cells

According to Table 1, researchers have used gelatin (collagen)^61^ and plastic without coating to expand TM cells. However the ECM of the human TM contains numerous structural and organizational components such as, laminins, collagens, elastin, fibronectin, fibrillins, proteoglycans, matricellular proteins, etc, (Reviewed in^63^), which more closely resembles Matrigel matrix, a solubilized basement membrane preparation containing laminin, collagen IV, heparin sulfate proteoglycans, entactin/nidogen and some growth factors.

### 6.1 MESCM+5% FBS as the culture medium for TM cells

Many cell culture media including DMEM+10% FBS,^64^ Medium 199E (originally designed for culture of chick embryo fibroblasts, a rich but not stem cell culture medium) + 20% FBS^61^ and IMEM (Improved Minimum Essential Medium, contains many components in MEM with addition of amino acids, vitamins and metabolites)+10%‐20% FBS^65^ are not stem cell culture media and are inappropriate for TM progenitor cell culture. A more optimal medium for TM cells is stem cell growth medium (SCGM)+5% FBS. However, this medium contains serum and addition of serum may not be required for culturing of TM cells. We have reported that the ability of LNCs in generating MSC when cultured in MESCM (a stem cell culture medium used in our lab) and supporting LEPC diminishes when they were expanded based on the conventional method, that is, on plastic in DMEM with 10% FBS.^66^

### 6.2 TM progenitors

One interesting report^62^ showed that passage 3 (P3) TM cells that were isolated by collagenase digestion on a non‐adhesive substrate in SCGM exhibited clonal growth and were multipotent including being able to be differentiated into adipose‐like cells. However, the authors cannot induce P3 TM cells into keratocyte‐like cells if cultured and expanded in SCGM. The authors claimed that if the cell aggregates were isolated by collagenase digestion and cultured directly in keratocyte differentiation medium (advanced MEM, 10 ng/ml bFGF, 0.1 mmol/L ascorbic acid), the cells will have detectable keratocan by RT‐PCR and immunostaining. Such a claim is questionable because the authors claimed the cells have detectable keratocan by RT‐PCR and immunostaining, but not the cells after passage. This raised the question that such induced “keratocytes” might actually come from contamination of keratocytes during isolation because contaminated keratocytes may be eliminated after a series passages (for example, the results from P3 passage TM cells in Du's case and in our results from P3 TM cells does not support such a claim). In fact, we found that TM cells did not express detectable keratocan transcripts by RT‐real‐time PCR and cannot be induced into keratocyte‐like cells even after the cells were cultured in 3‐D Matrigel.^53^ All Du's findings were that TM cells could be induced into adipose like cells.^62^ Therefore, it is important to thoroughly characterize TM progenitors using TM cell markers, embryo stem cell markers, and neural crest progenitor markers.

Since we can expand the TM cells successfully, we need to characterize these cells in our study. Previously, AQP1, MGP, CHI3L1, TIMP3 were used as TM markers.^62^ The water channel aquaporin 1 (AQP1) was found in the TM in vivo^67^ and in vitro, which has an important role in mediating aqueous out‐flow.^12^ MGP acts as a calcification inhibitor playing a key role in IOP homeostasis through mediating calcification in TM.^67^ CHI3L1 acts against cell death, ECM remodelling and inflammation. TIMP3 (Metalloproteinase inhibitor 3) acts as an inhibitor against the matrix metalloproteinases to degrade the extracellular matrix. Recently, AnkG was also defined as essential for generation of new neurons in the brain.^68^ In summary, normal TM markers should include AQP1, MGP, CHI3L1, TIMP3, AnkG, MUC1.

Because there is no description of ESC and NC markers present in TM cells, we refer our work associated with adult stem cells/progenitors in the limbal niche and CE. We have described LNC express ESC and NC markers such as ABCG2, KLF4, Nanog, Oct4, Rex1, Sox2, PDGFRβ and N‐cadherin^66^, ^69^, ^70^, ^71^, ^72^ and HCEC express ESC and NC markers such as cMyc, KLF4, Nanog, Nestin, Oct4, Rex1, Sox2, SSEA4, FOXD3, HNK1, MSX1, p75NTR, Sox9.^73^, ^74^ Therefore, TM cells have similar characteristics as LNC and HCEC progenitors.

## 7 REPROGRAMMING OF TM CELLS INTO THEIR PROGENITOR STATUS BY 3‐D MATRIGEL IS MEDIATED THROUGH CANONICAL BMP SIGNALLING

Although a considerable amount is known about the transcriptional networks that regulate ESCs, relatively little is known about the signalling pathways that integrate intrinsic and extrinsic cues to maintain the pluripotent state and control reprogramming. BMPs are an important family of morphogens that regulate cell fate decisions in stem cells.^75^ In the BMP pathway, ligand binding to the heterotetrameric complexes of type II and type I receptors leads to phosphorylation of receptor‐regulated R‐Smads 1, 5, and 8 that in turn bind to Smad4 and accumulate in the nucleus to regulate transcription.^76^ In mouse ES cells (mESCs), BMP signalling together with leukaemia inhibiting factor (LIF) signalling is important for maintaining the pluripotent state,^77^ whereas TGF‐β/Activin signalling is critical in human ESCs and mouse stem cells that are derived from the epiblast (EpiSC).^78^ We have previously reported that limbal niche cells can be reprogrammed on 3‐D Matrigel to their progenitors by canonical BMP signalling.^79^ Also, induced pluripotent stem cells (iPSCs) could be reprogrammed on 3‐D Matrigel.^80^, ^81^, ^82^

Trabecular meshwork (TM) cells can be reprogrammed into their progenitor status on 3‐D Matrigel, similar to reprogramming of induced pluripotent stem cells (iPSCs).^80^, ^81^, ^82^ Such a conclusion is supported by that fact that BMP inhibitor, Noggin, could abolish up‐regulation of BMP1, BMP2, BMP4, BMP6 and BMPR2,^53^ attenuate higher transcript expression of embryonic stem cell markers Oct4, Sox2, Nanog, Klf4 and SSEA4 and neural crest markers FoxD3, MSX1, Sox9, Sox10 and PDGFRβ,^53^ and block nuclear translocation of Oct4, Sox2, Nanog and pSmad1/5/8.^53^ These data collectively suggest that canonical BMP signalling is activated in TM cells on 3‐D Matrigel to reprogram TM cells into their progenitor status.

## 8 CURRENT STATUS OF CELL THERAPY IN ANIMAL OR CLINICAL STUDIES USING TM CELLS

Only until recently, two significant mouse models have been established for studies of TM cell therapies.^83^, ^84^ Zhu et al reported that aqueous humour out‐flow was restored following transplantation of iPSC‐derived TM cells in a transgenic mouse model of glaucoma.^84^ Their results suggest that transplantation of iPSC‐TM may restore out‐flow facility and IOP in aged mice. However, this model is not perfect because the expression of myocilin and calnexin is still elevated in transplanted eyes of the mice, suggesting that ER stress is not corrected in the TM. Jain et al demonstrated that CRISPR‐Cas9‐based treatment of myocilin‐associated glaucoma using myocilin mouse model of POAG.^83^ The problem for this model is that the authors use CRISPR‐Cas9‐based genome editing, which may result in unwanted genetic consequences. To our knowledge, no clinical trials with TM cell therapies have been reported. The key technical challenge is how to generate functional TM cells in vitro without change in the phenotype. We use the strategy of suitable substrate, for example, Matrigel, to reprogram TM cells to their progenitor status and try to get mature TM cells resembling those in vitro. We expect that after more thorough studies of the role of TM cells in the development of glaucoma, a cell‐based therapy will be established for effective clinical treatment of glaucoma in the future.

## 9 CONCLUSION

Trabecular meshwork cells contain adult stem cells which can be expanded in vitro without differentiation and differentiated into corneal endothelial cells, chondrocytes and adipocytes, but not keratocytes or osteocytes. Thus, these stem cells can be potentially deployed for cell‐based therapy for glaucoma, corneal endothelial dysfunction and other ophthalmic diseases in general. Therefore, it is pivotal for us to continue the studies of defining TM stem cell properties and transitioning these results to clinical applications.

## CONFLICT OF INTEREST

No conflict of interest is declared.
